# COVID-19 Pandemic Fatigue and Its Sociodemographic, Mental Health Status, and Perceived Causes: A Cross-Sectional Study Nearing the Transition to an Endemic Phase in Malaysia

**DOI:** 10.3390/ijerph20054476

**Published:** 2023-03-02

**Authors:** Mohd Radzniwan Abdul Rashid, Sharifah Najwa Syed Mohamad, Ahmad Izzat Ahmad Tajjudin, Nuruliza Roslan, Aida Jaffar, Fathima Begum Syed Mohideen, Faizul Helmi Addnan, Nizam Baharom, Muslimah Ithnin

**Affiliations:** 1 Faculty of Medicine and Health Science, Universiti Sains Islam Malaysia, Nilai 71800, Malaysia; 2 Islamic Science Institute, Universiti Sains Islam Malaysia, Nilai 71800, Malaysia; 3 Faculty of Medicine and Defence Health, Universiti Pertahanan Nasional Malaysia, Sungai Besi, Kuala Lumpur 57000, Malaysia; 4 Corporate Communications Unit, Ministry of Health Malaysia, Putrajaya 62000, Malaysia

**Keywords:** pandemic fatigue, COVID-19, depression, anxiety, stress

## Abstract

This study aimed to explore the socio-demographic characteristics, mental health status, and perceived causes of pandemic fatigue with COVID-19 pandemic fatigue among the general population of Malaysia. The data was collected online during the transition from the COVID-19 pandemic phase to the endemic phase in Malaysia from 1 to 30 April 2022. Sociodemographic data, Depression Anxiety Stress Scale-21 (DASS-21), perceived causes of pandemic fatigue, and the Fatigue Assessment Scale (FAS) were included in the survey. The chi-square test and a simple logistic regression analysis were used to identify predictors of pandemic fatigue. The completed survey (*N* = 775) included individuals aged 18 years or above [mean 31.98 (SD = 12.16)] from all states in Malaysia. Pandemic fatigue prevalence was 54.2%. Severe to extremely severe depression, anxiety, and stress symptoms were detected in 11.2%, 14.9%, and 9.1% of the participants, respectively. Younger age, non-Malay ethnicity, living alone, and higher income categories were significantly higher in the fatigued group. Higher DASS-21 scores on all domains were associated with higher FAS scores. Meanwhile, high scores for perceived tiredness from complying with the COVID-19 Standard Operating Procedure (SOP), perceived risk of infection from COVID-19, perceived hardship due to the pandemic, perceived public complacency during the pandemic, and perceived changes due to the pandemic were associated with a higher FAS score. This study provides valuable information for policymakers and mental health professionals worldwide on pandemic fatigue and its associated factors, including mental health status in Malaysia.

## 1. Introduction

Novel pneumonia caused by a coronavirus has been explicitly named as severe acute respiratory syndrome coronavirus 2 (SARS-CoV-2). However, it is known more generally as COVID-19, which was first reported in Wuhan, China, and is highly infectious [1]. Its primary clinical symptoms include fever, fatigue or myalgia, dry cough, shortness of breath, or difficulty breathing. The COVID-19 infection can vary between mild and severe diseases that may lead to multi-organ failure and death [2]. Because it is a fast-spreading infection, the World Health Organization (WHO) declared the COVID-19 outbreak a public health emergency of international concern (PHEICs) on 30 January 2020 and subsequently declared it as a pandemic on 11 March 2020. Previously, the PHEICs following a pandemic announced by the WHO were polio in 2014 and Ebola in 2018 [3,4,5].

To alleviate the COVID-19 pandemic, countries worldwide enacted unprecedented public health and social measures. The WHO has recommended protective measures in response to the COVID-19 pandemic, such as maintaining a physical distance of at least one meter away from each other, wearing a face mask, avoiding crowded or poorly ventilated areas, and frequent handwashing [6]. The Malaysian government took steps to incorporate the WHO recommendations mentioned above in order to contain the infection. Due to increasing cases in Malaysia in early 2020, the Malaysian government implemented the Movement Control Order (MCO) on the 18th of March 2020, imposing strict Standard Operating Procedures (SOPs) and establishing laws to ensure the people adhered to the SOP [7]. Studies estimated that closing and restricting high-exposure business places, public areas, and schools were perhaps the most effective public health interventions against COVID-19 in the absence of vaccines at the time [8]. 

As for the record in Malaysia, the reported number of new COVID-19 cases on 1 April 2022 was 17,476, with 30 deaths, and the total number of active cases was 206,881 [9], which was higher compared to the 1178 new COVID-19 cases and 6 deaths on the 1 April 2021 [9]. However, to strike a balance between lives and livelihoods, the rigorous MCO initially applied was loosened. Adjustments to the stringency of the MCO were conducted carefully through ongoing analyses of the available disease transmission indicators, such as the incidence rate, R-nought (R0), and vaccination coverage [10]. Eventually, on 8 March 2022, Malaysia’s Prime Minister declared that the country would begin the transition to the endemic phase and reopen its borders on 1 April 2022 [11]. Not long after that, after nearly two years of battling the COVID-19 pandemic, the government started lifting certain SOPs. For example, from 1 May 2022, wearing a mask was no longer compulsory in open spaces, but only limited in closed places such as in public transport and health care facilities. In addition, there was no more restriction on opening and closing business hours thus enabling business owners to observe business hours stipulated in their pre-pandemic business licenses. Other than that, the 50% capacity limit for activities involving large gatherings was removed, and interstate travel regardless of a person’s vaccination status was allowed [11]. 

As the pandemic continued, it was clearly observed that in general people were getting tired by the strict conditions imposed by the governments [12]. Even though the number of cases was reduced after a certain period of MCO implementation, the emergence of new variants of COVID-19 contributed to its further spread, producing several waves of infection [10,13]. Additionally, the cycle of closing and re-opening of the economic and education sectors at this stage contributed to the fatigue experienced by the Malaysian people [14]. A study also reported that people were feeling tired of the government’s social/physical distancing and SOPs [15]. Their health was reported to be affected (physically and mentally), causing burnout. Psychological burnout is a broad term being used to refer to subjective complaints of mental or physical stress in reaction to an external stressor, i.e., the workplace [16]. The external stressor in turn is defined as an experience that is outside the range of the usual human experience and that would be markedly distressing to almost anyone, encompassing a variety of signs and symptoms in the physical, emotional, behavioural, and cognitive domains. Although it is specifically designated as an occupational hazard, it is shown that the burnout phenomenon cannot be specifically restricted to occupations as prolonged, unresolvable stress which is the recognised cause of burnout is not limited to work alone (non-occupational burnt out) [17]. In fact, ‘burnout’ is not categorized as a medical diagnosis in the *Diagnostic and Statistical Manual of Mental Disorders, 5th Edition* [18].

The reported impact of tiredness on mental status was consistent with findings globally in which deterioration in mental health, including depression/depressive symptoms, anxiety, psychological distress, and poor sleep quality, was observed since the pandemic began [19]. A local study involving Malaysian adults by Dai et al. [20], which was conducted six weeks after the MCO was issued, found that the mental health, consisting of insomnia, anxiety, and depression, of the public, had worsened. To illustrate further, the past epidemics and current studies published on COVID-19 suggest the potential for numerous long-term psychological effects during and following epidemics and the COVID-19 pandemic itself [21]. For example, in 2015, there was an outbreak of Middle Eastern Respiratory Syndrome (MERS) in Korea, resulting in a 20% mortality rate [22]. During the MERS epidemic, eight in ten of the general public reported fear of being infected, and about one in two reported having emotional distress [23]. Meanwhile, the 2014 Ebola outbreak caused unprecedented panic levels due to the virus’s extremely contagious and deadly characteristics, fast spread, and high mortality rate [24]. As for the COVID-19 pandemic, a systematic review by Zeng et al. [25] reports that after recovery from acute COVID-19, half of the survivors still have a high burden of either physical or mental sequelae for up to at least 12 months. In another finding, in the midst of the COVID-19 pandemic, a 12-month longitudinal study found that half of the respondents experienced COVID-19-related psychological distress, which had a long-term impact on their mental health [26].

In regard to the important mental health issues that have been discussed due to the COVID-19 pandemic [27,28,29], one may wonder if we face pandemic fatigue. The WHO defines pandemic fatigue as distress that can result in demotivation to follow the recommended protective behaviours, emerging gradually over time and being affected by a few emotions, experiences, and perceptions [30]. Pandemic fatigue can occur when people get tired of the pandemic measures and become less likely to follow public health practices or begin to drown out those messages [31]. In other words, pandemic fatigue has decreased the efficacy of populations complying with public health measures such as masks and social distancing. 

The risk factors for developing pandemic fatigue have been investigated as well. Studies found that pandemic fatigue was linked to being younger, having been previously infected with COVID-19, and having a low perceived severity of COVID-19 among others [31,32]. Meanwhile, a study by Yan et al. [33] reported that 25.7% of the participants were feeling physically and psychologically fatigued, and their association was fully mediated by self-perceived disruptions of COVID-19-related restrictions in daily life.

Even with the arrival of vaccines for COVID-19 in late December 2020 and the beginning of mass vaccinations in many countries, including Malaysia, the people still needed to adhere to the strict SOP implemented by the government, including self-control measures and personal hygiene. This is due to the emergence of the COVID-19 variant of concern (VOC) and low immunity over time, particularly among the elderly, immuno-compromised individuals, and vulnerable groups [10]. Additionally, while living with COVID-19, preventive measures and behavioural control were crucial. Nevertheless, pandemic fatigue negatively impacts individual feelings, thoughts, and behaviours [30]. The WHO warned that “COVID-19 fatigue” is growing across Europe as more people feel apathetic about a pandemic that has “exhausted all of us”. It evolves gradually with time, and many factors were found to affect it, such as each country’s culture, society, structure, and policy [34]. Nonetheless, data on pandemic fatigue are lacking in Asia, including Malaysia.

This conducted study on pandemic fatigue in Malaysia remains scarce and, to the best of our knowledge, no previous research in Malaysia had assessed the association between mental health and perceived causes of pandemic fatigue. Nevertheless, there are vital factors that might influence public pandemic fatigue. This study was carried out at the beginning of the transition to the endemic phase to understand whether the relaxation of restrictions imposed during the pandemic would reduce pandemic fatigue among Malaysian populations. In our study, we utilized the translated Malay version of the Fatigue Assessment Scale (FAS) to assess pandemic fatigue. Its original English version has been proven useful and had been used to assess pandemic fatigue in previous studies [35,36]. Apart from that, the perceived cause of pandemic fatigue was assessed by a newly developed scale and mental health was examined by a 21-item Depression, Anxiety, and Stress Scale (DASS-21) [37]. These study tools are described in the materials and methods section. Understanding the factors essential for pandemic fatigue is critical for implementing behavioural mitigation strategies as health behaviours using the psychology of health behaviour change to gain control of the latest surge [30]. We have sought to address this gap by conducting a study to investigate the prevalence of COVID-19 pandemic fatigue among the general Malaysian population and its association with sociodemographic, mental health, and perceived causes of pandemic fatigue. 

### Research Questions

The main research questions guiding this study were as follows:

1. What is the COVID-19 pandemic fatigue prevalence among Malaysians?
2. What are the mental health status and perceived causes of pandemic fatigue in Malaysia?
3. What is the association between sociodemographic, mental health, and perceived causes of pandemic fatigue with pandemic fatigue?

## 2. Materials and Methods

### 2.1. Study Design and Settings

This study was a cross-sectional online survey in Malaysia. The subject recruitment and data collection were conducted online for four weeks nationwide across Malaysia from 1 April 2022 until 30 April 2022. All included subjects were of Malaysian nationality, above 18 years old, and residing in Peninsular Malaysia, including Sabah and Sarawak. Social media platforms, including WhatsApp^®^, Facebook^®^, and personal emails, were used to distribute the Google Form questionnaires. Google forms were used to get information as this method was deemed appropriate for collecting data during the pandemic. The technique ensures no physical human contact, and the data collected via Google Forms were secured with a complex password and created using an account specific to this study. The study was approved by the ethical committee of Universiti Sains Islam Malaysia (USIM), Ethics Committee/IRB Ref No: USIM/JKEP/2021-178.

The subjects were invited to participate using a convenient sampling technique through these multiple social media platforms. They were requested to pass the invitation on to their contacts. The estimated time to complete the survey was around 10 to 15 min.

Sample sizes in our study were in accordance with the guidelines of Krejcie and Morgan [38]:

$$n=\frac{X^{2}NP\left(1-P\right)}{\left[ME^{2}(N-1\right)]+[X^{2}P\left(1-P\right)]}$$

where *n* = sample size,

*X*^2^ = critical value at 95% confidence interval,

*N* = population size,

*P* = sample proportion, 

and *ME* = margin error.

According to this method, if the total population is more than a million, the minimum number of participants required is 384 (95% confidence level with a 5% error estimate). To overcome withdrawal sampling/insufficient responses/missing data, we aimed to recruit more than 768 participants (double the minimum required number). In the study, we managed to recruit a total of 775 participants, which was sufficient for the purpose of the study. 

### 2.2. Measurement of Sociodemographic Characteristics

Data on age (years), gender, ethnicity, state, educational background, living arrangements, household income, list of chronic diseases, history of positive COVID-19, the status of the working station during the pandemic, and front-line status were collected as sociodemographic variables. 

Gender was either male or female. Ethnicity selection was of Malay, Chinese, Indian, or others, including Sabahan or Sarawakian. Malaysia’s states included the states from the North Peninsular (Perlis, Kedah, Penang, and Perak), the Central Peninsular (Selangor, Kuala Lumpur, and Putrajaya), the South Peninsular (Negeri Sembilan, Melaka, and Johor), the East Peninsular (Pahang, Terengganu, and Kelantan), and Borneo (Sabah, Sarawak, and Labuan). The educational background was divided into ‘secondary’ and ‘tertiary’, and living arrangements into ‘alone’ and ‘living with spouse/family members. The monthly household income (RM) was classified as low income of ≤RM4850 (≤US$1094), and middle or high income of more than RM4850 (US$1094). Those with any chronic illnesses were categorized as having one or more illnesses. Whether participants had been infected with COVID-19 viruses, have an occupation that required working from home, or were working or fighting COVID-19 at the front line were data gathered during the data collection. 

### 2.3. Measurement of Pandemic Fatigue

The Fatigue Assessment Scale (FAS) was used to measure the fatigue level of the respondents [39]. The classification for fatigued and non-fatigued groups was done in accordance with the scoring protocol given by the Fatigue Assessment Scale (FAS) developmental team. The FAS is a 10-item general fatigue questionnaire to assess fatigue. Five questions reflect physical fatigue and five questions (questions 3 and 6–9) mental fatigue [39,40].

The response options for every item were five options on a Likert scale: 1 (Never), 2 (Rarely), 3 (Sometimes), 4 (Always), and 5 (Often). An answer to every question had to be given, even if the person did not have any complaints at that moment. Scores on questions 4 and 10 needed to be recorded (1 = 5, 2 = 4, 3 = 3, 4 = 2, 5 = 1). Subsequently, the total FAS score could be calculated by summing the scores on all questions (recoded scores for questions 4 and 10). The sum of the FAS scores ranged from 10 to 50. A total FAS score of less than 22 indicates no fatigue, and a score of 22 and more indicates fatigue.

The original FAS was translated into the Malay language (after permission was granted from the original author) using a translation and back translation technique, where an expert panel assessed the content validity. First, two appointed independent language experts translated the English versions into Malay. Then, it was back-translated into English by two appointed independent language experts. The back-translated versions were then compared with the original FAS to ensure the accuracy of the FAS-BM. An expert panel consisting of a public health physician, a psychiatrist, and a family medicine specialist reviewed the translated FAS-BM. It was assessed sentence-by-sentence to ensure the translation’s accuracy, the instruction’s comprehensibility, and the cultural relevance for it to be applied in the local setting. Several changes had been made to ensure that the tool was culturally relevant. The pre-final version of FAS-BM was piloted. The internal consistency of the Malay-translated FAS was 0.88 (Cronbach’s alpha), indicating good reliability.

### 2.4. Measurement of Mental Health

The 21-item Depression, Anxiety, and Stress Scale (DASS-21) is a self-reporting questionnaire for assessing depression, anxiety, and stress symptoms [37]. Seven items represent each emotional state. The Malay version of the questionnaire has been verified in the general Malaysian population, with a Cronbach’s alpha of 0.84, 0.74, and 0.79, respectively [40], and this form was used in the study. 

The participants were asked to rate the severity of their symptoms throughout the previous week, and the scores for each subscale were calculated using data from a prior study. Normal (0–9), mild depression (10–12), moderate depression (13–20), severe depression (21–27), and extremely severe depression (21–27) were the depression scale scores (28–42). Normal (0–6), mild anxiety (7–9), moderate anxiety (10–14), severe anxiety (15–19), and extremely severe anxiety were the anxiety scale scores (20–42). Normal (0–10), mild stress (11–18), moderate stress (19–26), severe stress (27–34), and extremely severe stress were the stress scale scores (35–42).

### 2.5. Measurement of Perceived Causes of Pandemic Fatigue

From the literature review, media, and brief interviews with the public, 38 items were constructed as the perceived cause of pandemic fatigue. Face and content validity was conducted with a panel of four experts, and six items were removed. A pre-test survey with 250 respondents was conducted for the 32 items, and an exploratory factor analysis was performed. Five domains were suggested as causes of pandemic fatigue, with four items removed: (1) perception of fatigue complying with COVID-19’s SOP, (2) perception of risk of infection with COVID-19, (3) perception of distress due to the pandemic, (4) perception of negligence toward pandemic, and (5) perception of change due to the pandemic. Another survey was conducted with 227 respondents, and a confirmatory factor analysis was performed. The final version consisted of (1) perception of fatigue complying with COVID-19’s SOP (9 items), (2) perception of risk of infection with COVID-19 (4 items), (3) perception of distress due to the pandemic (4 items), (4) perception of negligence toward pandemic (3 items), and (5) perception of change due to pandemic (3 items), with a total of 23 items.

Each item had 5 points Likert scale responses: never felt (1), some days in a month (2), less than 3 times in a week (3), more than 3 times in a week (4), and felt every day (5). The sum of all scores in each domain was calculated. The normality of the score distribution of each domain was checked. The perceived causes scores were then dichotomized into two categories. Those who scored below the mean score were categorized as having low perception levels for the domain, and respondents scoring greater than or equal to the mean score were categorized as having a high perception level. 

### 2.6. Statistical Analysis

Data cleaning was conducted before analysis to detect and remove any redundant records. For example, if the same respondent filled out the questionnaire multiple times, this can be recognised through the respondent’s email address, so the duplicate data set was deleted from the data. Verification of the response was also conducted to guarantee that the same answer was given only once.

Descriptive statistics were used to analyse the sociodemographic characteristics, the mental health status, and the responses to questions concerning perceptions of pandemic fatigue. Frequencies and percentages were used to present categorical variables. The demographic and job variables were described with a mean (SD), number (*n*), and percentage (%). 

A graphical technique was used to assess whether a data set was approximately normally distributed. If the histogram graphs of frequency data were approximately showing a bell-shaped and symmetric form around the mean, then the data was assumed to be normal. 

Simple logistic regression was conducted to determine the association of continuous variables of age with pandemic fatigue. While Pearson’s chi-square test was conducted to determine the association between the respondents’ categorical characteristics and pandemic fatigue. Continuous mental health variables and a perceived cause of pandemic fatigue associated with pandemic fatigue were analysed with simple logistic regression. Statistical analysis was done using the Statistical Package for the Social Sciences (SPSS, Chicago, IL, USA) (version 24.0; IBM Corp, Armonk, NY, USA). *p* < 0.05 was taken as a cut point for statistically significant results.

## 3. Results

### 3.1. Description of Pandemic Fatigue, Mental Health Status, and Perceived Causes of Pandemic Fatigue

A total of 775 respondents across Malaysia participated in the survey. The mean (SD) age was 31.98 (12.16). The mean (SD) for the pandemic fatigue score was 23.72 (7.98). The overall prevalence of pandemic fatigue was 54.2% (*n* = 420), whereas 45.8% (*n* = 355) were in the non-fatigued group.

**Figure 1:**
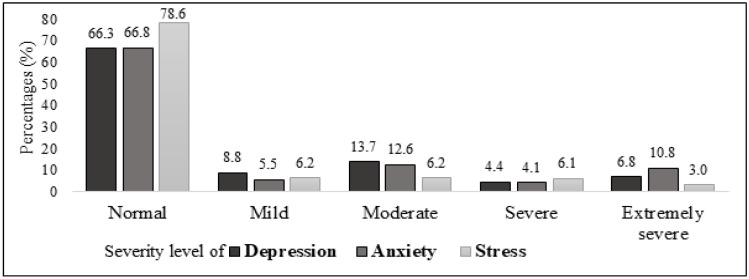
Level of depression, anxiety, and stress among all respondents (*n* = 775).

The mean (SD) scores for depression, anxiety, and stress were 7.91 (9.71), 6.39 (8.46), and 8.58 (9.62), respectively. Figure 1 shows the participants’ depression, anxiety, and stress by categories. The prevalence of severe to extremely severe depression symptoms was 11.2% (4.4% severe and 6.8% extremely severe). Moreover, 14.9% of the respondents presented severe to extremely severe anxiety symptoms. Meanwhile, the prevalence of severe to extremely severe depression symptoms was 9.1%.

The mean (SD) scores for each domain for perceived causes of pandemic fatigue are as follows: perceived tiredness from complying with the COVID-19 SOP (3.42, 0.86), perceived risk of infection from COVID-19 (3.76, 0.84), perceived hardship due to the pandemic (2.81, 0.86), perceived public complacency during the pandemic (3.16, 0.89), and perceived changes due to the pandemic (3.49, 0.88).

For the perception of fatigue to comply with the SOP of COVID-19, 63.6% of the respondents agreed and strongly agreed that the cause of their pandemic fatigue was that they must obey (MCO). While 61.3% of the respondents agreed and strongly agreed that having to test for COVID-19 using an RTK/PCR test when indicated was the cause of their pandemic fatigue. 

For the perceived risk of infection with COVID-19, 70.8% of the respondents agreed and strongly agreed that they were worried about the emergence of a dangerous new variant of concern. In addition, 67.1% had negative perceptions when hearing about the daily cases and deaths of COVID-19. About half of the respondents disagreed and strongly disagreed that they had distress due to job insecurity or loss of job (45.5%). Meanwhile, more than half of the respondents (56.5%) disagreed or strongly disagreed that they experienced a lack of support from family and friends. 

**Figure 2:**
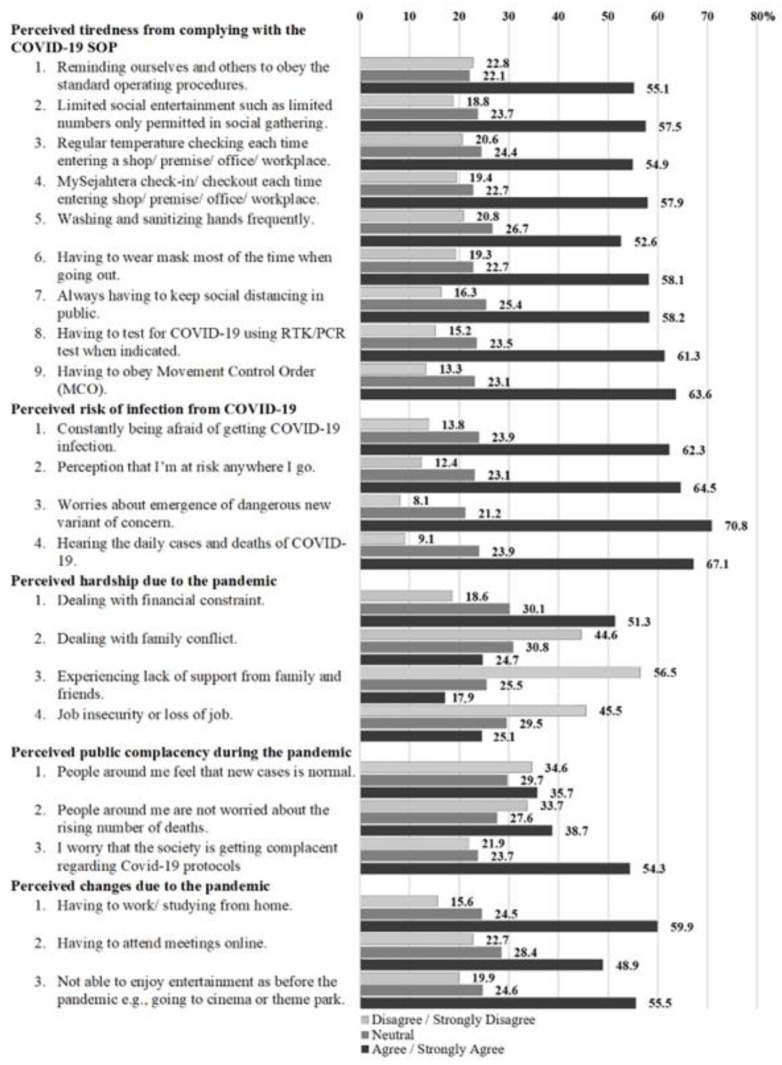
Perceived causes of pandemic fatigue—domain and individual items—among respondents (*n* = 775).

For the perception of negligence towards the pandemic, more than half of the respondents (54.3%) were worried that society was getting complacent regarding COVID-19 protocols. Finally, 59.9% of the respondents agreed and strongly agreed that having to work/study from home was the perceived cause of pandemic fatigue. More than half (55.5%) also agreed and strongly agreed that not being able to enjoy entertainment as before the pandemic was the cause of pandemic fatigue. The details for each statement are shown in Figure 2.

### 3.2. Association between Sociodemographic Characteristics and Pandemic Fatigue

The results of the univariate analyses of the association between sociodemographic characteristics and pandemic fatigue are shown in Table 1. There were significant differences (all *p* < 0.05) between the non-fatigued and fatigued groups regarding age, ethnicity, living arrangement, and income categories. A logistic regression was performed to ascertain the effects of age on the likelihood that participants have pandemic fatigue. An increase in age was associated with a reduction in the likelihood of exhibiting pandemic fatigue (OR = 0.967, 95% CI = 0.956, 0.979, *p* < 0.001). Non-Malay ethnicity was more significant in the fatigue group (*p* = 0.021). Those who lived alone indicated higher fatigue levels (*p* < 0.001), while participants with higher incomes tended to be fatigued (*p* < 0.001).

### 3.3. Association between Mental Health and Pandemic Fatigue

The association between mental health scores and the fatigue and non-fatigue categories is shown in Table 2. A simple logistic regression was performed to ascertain the depression, anxiety, and stress scores on the likelihood that participants would have pandemic fatigue. An increase in the depression score was associated with an increased likelihood of having pandemic fatigue (OR = 1.29, 95% CI = 1.24, 1.34, *p* < 0.001). An increase in the anxiety score was associated with an increased likelihood of having pandemic fatigue (OR = 1.27, 95% CI = 1.22, 1.32, *p* < 0.001). Finally, for the stress domain, an increase in the stress score was associated with an increased likelihood of having pandemic fatigue (OR = 1.23, 95% CI = 1.12, 1.27, *p* < 0.001).

### 3.4. Association between Perceived Causes of Pandemic Fatigue and Fatigue Category

The results of the analysis of perceived causes and factors influencing pandemic fatigue are displayed in Table 3. An increase in the score for the perception of fatigue to comply with the SOP of COVID-19 was associated with an increased likelihood of having pandemic fatigue (OR = 1.02, 95% CI = 1.00, 1.04, *p* < 0.001). Then, an increase in the score for the perceived risk of infection with COVID-19 was associated with an increased likelihood of having pandemic fatigue (OR = 1.06, 95% CI = 1.02, 1.11, *p* = 0.008). For the perception of distress due to the pandemic, an increase in the score was associated with an increased likelihood of having pandemic fatigue (OR = 1.17, 95% CI = 1.12, 1.22, *p* < 0.001). Meanwhile, an increased score in the perception of negligence toward the pandemic was associated with an increased likelihood of pandemic fatigue (OR = 1.10, 95% CI = 1.05, 1.17, *p* < 0.001). Finally, an increase in the perception of change due to the pandemic was associated with an increased likelihood of having pandemic fatigue (OR = 1.12, 95% CI = 1.06, 1.18, *p* < 0.001).

## 4. Discussion

It has been more than a year since the COVID-19 pandemic hit globally. People worldwide, including Malaysians, have been complying with various policies enacted by governments to curb the spread of COVID-19 infection [7,44]. To the best of our knowledge, this is the first study conducted among the Malaysian population on the prevalence and the perceived causes of pandemic fatigue. Most research on pandemic fatigue reported that the phenomenon needed to be identified and managed effectively. This is because it is feared that the incidence of COVID-19 could continue to rise, resulting in a surge of new COVID-19 variants. This is the worst concern of pandemic fatigue because people are bored and tired of following the implemented SOPs to combat the pandemic. Ultimately, the plan to see a decline in infected cases seemed less likely. 

Our current study revealed that one in two Malaysians (54.2%) experience pandemic fatigue. This finding was relatively lower than in a study done in Turkey, where their pandemic fatigue score was 64.1% [45]. The comparison is reasonable as they used a similar tool to assess fatigue: Fatigue Assessment Scale (FAS). Nevertheless, it is similar to studies conducted among populations of other countries whereby the prevalence of pandemic fatigue was 56.4% among the population in Turkey [46], 43.7% among the population in Hong Kong [47], and 49.0% in Xi’an, China [48]. The slight variation in the prevalence of pandemic fatigue may be because their studies were conducted at the beginning of the pandemic when most people were less informed about the COVID-19 pandemic.

Mental health difficulties during the COVID-19 pandemic were reported to be higher than in pre-pandemic days, and this is similar worldwide. The COVID-19 Mental Disorders Collaborators concluded that there was an increment of 27.6% in cases of major depressive disorder, with a 25.6% increment in cases of anxiety reported worldwide [49]. A comparison study between the United Kingdom (UK) and Germany demonstrated that 25% of both responders reported a worsening of the general psychological symptoms, and 20–50% of them reached the clinical cut-off for depressive and dysthymic symptoms as well as anxiety [50]. An exceptionally large study among the community in China involving larger provinces, with 56,679 participants across all 34 province-level regions in China, reported daunting results, whereby 27.9% of participants had symptoms of depression, 31.6% had symptoms of anxiety, 29.2% had symptoms of insomnia, and 24.4% had symptoms of acute stress during the outbreak [51]. In our study, the prevalence of depression, anxiety, and stress were 33.5%, 33.1%, and 21.4%, respectively, which is similar to the above studies. 

Another important finding was that the prevalence of severe to extremely severe depression, anxiety, and stress symptoms were 11.2%, 14.9%, and 9.1%, respectively. The alarming figures should trigger health authorities to tackle the problem immediately, as prevention is better than cure. Creating awareness and implementing an early intervention is particularly important in helping patients who exhibit early-onset symptoms, as it may prevent them from getting severe morbidity. Unfortunately, the rising incidence of COVID-19 during the pandemic has depleted mental health resources worldwide. This exposed the gap in the funding of mental health services globally. Many health assistance and facilities for healing mental health have been implicated.

There were multiple factors for the increment in mental health status: (1) the inability to move freely due to COVID-19 restrictions, (2) the need for home confinement to restrict movement, (3) difficulty in obtaining mental health assistance, (4) economic turmoil due to sudden economic shutdown, and (5) other direct COVID-19-related factors, such as fear of oneself or family members contracting COVID-19 and the difficulties in obtaining face masks during the initial phase of the pandemic. The fact that job losses were apparent during the pandemic had a significant impact, whereby job loss is a known associated factor for depression, anxiety, distress, and low self-esteem; importantly, job losses may lead to higher rates of substance use disorder and suicide. In Malaysia, the COVID-19 pandemic has affected the labour market and led to an increment in unemployment. It has been reported that the unemployment for the year 2020 was 711,000, which is an increase from 508,200 for the year 2019 and brought the unemployment rate to 4.5% (2020) from 3.3% (2019), as reported by the Department of Statistics, Malaysia [52]. In addition, those who lost their job had higher rates of symptoms of mental illness than those without jobs or income loss (53% vs. 32%). The job issue in our study reflected this problem when more than half of the respondents felt distressed due to job insecurities or from the actual loss of employment.

Compared to local Malaysian research conducted between 12 May and 5 September 2020, around two months after the pandemic’s commencement, their findings were higher, with the prevalence of depression, anxiety, and stress being 59.2%, 55.1%, and 30.6%, respectively [53]. Our research was conducted when the Malaysian government planned to transition from the pandemic to the endemic phase to strike a better balance between economic, social, and health implications. The transition was started by lifting a few restrictions on 1 April 2022. At the time, mask-wearing became less strict and it was only mandated in closed confined buildings and crowded areas, as well as in public transport [54]. As our study was conducted at the beginning of this transition phase, we observed a slight decline in psychological morbidity compared to that local study. At that moment, people began to have hope that the pandemic could reach the end of the tunnel. 

Our present study demonstrated that the respondents felt fatigued in the following domains of perceived causes of pandemic fatigue in decreasing order: perception of change due to the pandemic (52.8%), a high perception of risk of infection with COVID-19 (51.7%), and a high perception of distress due to the pandemic (49.2%) with a high perception of negligence toward the pandemic (48.4%). Interestingly, we found the least high perception of fatigue to comply with the SOP of COVID-19 (45.4%). Nonetheless, our findings showed that more than half of the respondents agreed that complying with the MCO causes fatigue. In Malaysia, the strict MCO laws were implemented immediately on 18 March 2020, after much deliberation on their negative effect on the economy [7,55]. Other crucial steps imposed were the requirement for face masks to be always worn outside the house, hand sanitizing, recording before entering the premises either with a QR code or manually, and physical distancing. The enforcement of the MCO has caused tremendous chaos and had the most impact on the economy and social well-being of the citizens [56]. 

The fear of contracting the COVID-19 infection is confirmed and is a potential predictor of pandemic fatigue [57]. Most people practice the SOP to potentially reduce their risk of infection as many understand the severe consequences of an infection that could cause death. Hence, various authority measures to mitigate this issue have led people to experience fatigue as they must be on guard 24 h a day. Death anxiety is a more accurate term to describe the situation. It is a psychological state arising from one’s fear of death or being harmed [58,59]. When this is persistently present in one’s mind, it could lead to mental fatigue. The prevalence of death anxiety has been reported in a few studies and discussed extensively regarding its detrimental consequences [60,61]. For example, a Lebanese study reported that 33.7% of their respondents feared COVID-19 [62]. While a Hong Kong study reported that 58.7% of respondents had a high fear of COVID-19 with a high anxiety level of 60.4% [47]. However, a Malaysian study investigating the fear of COVID-19 noted lower results, where only a quarter of the respondents (27.1%) reported elevated levels of fear of COVID-19 [60]. In that respect, our study found the prevalence of anxiety to be 33.1%, amounting to a third of the respondents. Our study also reported that 67.1% had negative perceptions when hearing about the daily cases and deaths of COVID-19. It is important to note that the previous study highlighted the fear of infection and how restrictions for prevention, such as isolation, could exacerbate those with pre-existing psychological distress, and worsen the condition [63]. 

Good social support is one of the fundamentals of optimum health. It is increasingly evident that this element is positively related to human mental health and quality of life [64,65]. Unfortunately, some of the literature reports that during the pandemic people received less social support for multiple reasons and the strict laws on social distancing, quarantine, or self-isolation are not exceptional [66,67,68]. However, this finding contradicts previous studies as more than half of the respondents disagreed with receiving a lack of support from family and friends. Similarly, a Jordanian study found that the assessment of social support indicated a moderate-to-high level of perceived support from family, friends, and significant others [68]. In addition, a Hong Kong study revealed that family well-being and communication quality are essential factors in fatigue prevention [48]. 

Another aspect that led to the respondents’ fatigue was having to test for COVID-19 whenever indicated. Many countries implemented this step, especially when borders were opened and traveling was allowed for some countries, including Malaysia [69]. Other instances were to prove that they tested negative for COVID-19 before joining large gatherings, such as weddings and conferences. Apart from the psychological distress that the regulations would instill, it would also be a financial burden as the test must be paid for out-of-pocket. During the initial period, the cost of a COVID-19 PCR test was relatively exorbitant, which the low-income group would need help paying. Fortunately, by realizing this obstacle, the Malaysian government instructed kit test manufacturers to reduce the price to the most affordable one and eventually decided for rapid test kits (RTK) to be allowed for screening at a much lower price than PCR tests [70,71]. This study revealed that 61.3% of respondents agreed or strongly agreed that having to test for COVID-19 using RTK/PCR tests when indicated was a cause of pandemic fatigue.

Our study revealed that 70.8% of the respondents agreed or strongly agreed that they were worried about the emergence of a dangerously new COVID-19 variant known as variants of concern (VOC). The emergence of VOC is expected to occur if herd immunity in the community has not been achieved in a given period of time [72]. For example, the deadly Delta variant associated with severe morbidity and mortality that emerged during the third wave in Malaysia was immediately feared by the people in the country [73]. Later, the emergence of the Omicron variant stirred Malaysia and the world, indirectly signaling that the pandemic was never going to end [74]. 

Working from home had become a new norm during the pandemic. It was a tremendous change in the working environment and posed great challenges to everyone. Working from home has provided advantages and disadvantages for both employees and organizations [75]. Schools were also instructed to conduct virtual classes, despite some states and regions having poor internet connectivity [76]. Our study revealed that more than half stated having to work or study from home was the cause of their pandemic fatigue.

Our sample population feared that society was getting complacent regarding COVID-19 protocols. A prior study reported a gradual reduction in adherence to protective behaviours against COVID-19 from March through December 2020, as hypothesized in expectations of fatigue [77]. There have been a number of research focusing on adherence to and compliance with COVID-19 measures. One is a notable study in Belgium with a sample of 2008 participants [78]. The study found that the measure of wearing a face mask while restricting their social bubble (to five people or less) was the one that was being followed least. 

Compliance with restrictions was strongly influenced by the perceived usefulness of the measures and perceived personal capacity to adhere. Therefore, the author suggested that informing people of the hazardous risks and complications of COVID-19 could be an effective intervention to ensure that a high level of compliance remained. Another study, predicting compliance to COVID-19 sanitary measures (handwashing, mask-wearing, physical distancing, and social distancing), found that persons who were living alone, female gender, later in the academic curriculum, having higher general and health anxiety, higher academic involvement, and higher risk perception were positively associated with adherence to the COVID-19 measures mentioned earlier [78]. An Indonesian study with 461 adults looked at social determinants of COVID-19 protocol adherence and reported gender, age, educational level, economics, and social status were determinants of health protocol adherence [79]. Thus, we can conclude that social determinants play a principal factor in determining COVID-19 adherence and intervention should not only focus on this aspect but also on promoting and educating the public that risks and complications from COVID-19 are vitally important. 

Our study demonstrated that younger adults were significantly associated with fatigue. Several other studies have found that the younger age group was at risk of pandemic fatigue [31,46,47]; it was reported that younger age was associated with higher internet usage [80,81]. Therefore, we could conclude that they are more exposed to mixed and misleading information about COVID-19, which is spreading rapidly through the internet. The subsequent abundant information could lead to them being fatigued due to information overload. Not only that, but the younger age group also faced issues such as adjusting to working from home or having children who have to be schooled from home, thus requiring greater internet usage. The same applies to university or college lecturers and students who suddenly had to change their teaching and learning processes to online classes and exams [82]. This caused them to feel tired physically and mentally, leading to fatigue because of the pandemic, not to mention other challenges such as poor internet connection, lack of focus, and non-conducive situations [83].

The COVID-19 pandemic has had a substantial impact on household income. According to the report by DOSM [52], in 2020, the mean monthly household gross income in Malaysia dropped from 2019 to 2020 by 10.3%. As a result, some households experienced a decline in income, with a considerable number of them drifting from the higher-income to the lower-income categories. In addition, the higher-income group may have a higher financial commitment in life, such as paying their credit cards and loans, as they are more likely to be granted bank loans. This potentially causes more distress due to income reduction and the uncertainty of the pandemic period. Our study revealed that higher-income groups were significantly associated with pandemic fatigue. Perhaps a sudden change brought about by the pandemic made this income group tired and frustrated. This is in line with a study that reported the top income group had experienced burnout and fatigue due to the pandemic [84]. 

Furthermore, this study also revealed that staying alone and having concurrent psychological morbidity were other important factors contributing to pandemic fatigue. Staying alone poses a higher risk of distress due to a lack of social support. As mentioned earlier, the social support system is an integral component in protecting oneself from psychological distress during the COVID-19 pandemic [85]. Meanwhile, it cannot be denied that pandemic fatigue is strongly associated with fear, fatigue, and worsening of psychological problems if it is not identified early. Hence, the government must ensure that mental health facilities are accessible to all. Due to this, the Malaysian government announced an increase in the mental health budget allocation for 2023 [86]. 

With this result, we suggest that during the pandemic, anyone attending a healthcare center should be asked if they feel fatigued. Nonetheless, the identification of the problem should be pursued not only pertaining to the COVID-19 pandemic but to any other type of pandemic should it arise. As our study found that those of younger age, non-Malay ethnicity, living alone, and in the higher income categories, including those suffering from psychological morbidity, were at an increased chance of getting pandemic fatigue, screening these individuals are highly recommended in Malaysia. The importance of early identification and proper management of pandemic fatigue must be considered as the likelihood of having another pandemic is still possible. In addition, it is recommended that further studies be conducted during the endemic phase to see if the fatigue persists although the stringent law to fight COVID-19 infection has been lifted. 

The strength of this study is that it extends the field of research on pandemic fatigue to mental health and the perceived causes of pandemic fatigue among the Malaysian population, which is in line with the recommendation by the WHO. In addition, this was a nationwide study involving all states in Malaysia. Finally, the study period in which Malaysia was at the transition to the endemic phase is unique as it gives valuable insights on post pandemic fatigue and its causes.

Some limitations need to be explained in this study. First, this study was cross-sectional and any causal-relationship conclusions could not be drawn among the variables in this study. Longitudinal research should be conducted in the future to address this limitation. Secondly, this survey was performed via internet platforms, which may cause some response bias. Thirdly, Malaysia is a multiracial country; hence, the finding may not reflect the opinion of all ethnic groups residing in the country. Nevertheless, this study provided results that suggest pandemic fatigue may have a more significant effect on a particular ethnic group, which suggests future targeted research and intervention in reducing the impact of pandemic fatigue in this group. Finally, the study duration in Malaysia was during the transition to the endemic phase, enabling a description of the trend and pattern regarding pandemic fatigue and its perceived causes.

## 5. Conclusions

Pandemic fatigue exists, and its prevalence remains high. Although the data were collected during the transition period to the endemic phase in Malaysia, its tremendous impacts on mental health, social well-being, and the economy can still be observed. There was an association between pandemic fatigue and younger age, ethnicity, employment, income, staying alone, home, and psychological morbidity. In addition, the perceived causes, including tiredness from complying with the COVID-19 SOP, perceived risk of infection from COVID-19, perceived hardship due to the pandemic, perceived public complacency during the pandemic, and perceived changes due to the pandemic, were associated with a higher score of pandemic fatigue. Therefore, the authors suggest that further study is recommended at some point in the endemic period to see if the fatigue persists at a time during which laws and regulations to combat the COVID-19 infection have been lifted.

## Figures and Tables

**Table 1:** Association between socio-demographic characteristics and non-fatigued and fatigued groups.

Sociodemographic Characteristics		Official Demographic Data (Million) ^a^	Total (*N*) (100%)	Fatigued ^b^	Non-Fatigued	Statistic
				(*n* = 420), *n* (%)	(*n* = 355), *n* (%)	
Age, mean (standard deviation)				29.78 (11.115)	34.6	OR = 0.967 [95% CI = 0.956, 0.979], *p* < 0.001 *
					(12.831)	
Gender	Male	17 [41]	268	133 (49.6)	135 (50.4)	χ^2^ = 3.44, *p* = 0.064
	Female	15.7 [41]	507	287 (56.6)	220 (43.4)	
Ethnicity	Malay	69.9 [41]	637	333 (52.3)	304 (47.7)	χ^2^ = 5.30, *p* = 0.021 *
	Non-Malay	30.1 [41]	138	87 (63.0)	51 (37.0)	
Regions in Malaysia	North Peninsular		91	53 (58.2)	38 (41.8)	χ^2^ = 3.65, *p* = 0.456
	Central Peninsular		267	154 (57.7)	113 (42.3)	
	South Peninsular		216	112 (51.9)	104 (48.1)	
	East Peninsular		161	81 (50.3)	80 (49.7)	
	East Malaysia		40	20 (50.0)	20 (50.0)	
Education level	Secondary		63	39 (61.9)	24 (38.1)	χ^2^ = 1.64, *p* = 0.200
	Tertiary		712	381 (53.5)	331 (46.5)	
Living arrangement	Alone		412	250 (60.7)	162 (39.3)	χ^2^ = 14.91, *p* < 0.001 *
	With family/spouse		363	170 (46.8)	193 (53.2)	
Income category	Low (≤MYR4850)	2.91 [42]	303	189 (62.4)	114 (37.6)	χ^2^ = 13.42, *p* < 0.001 *
	Middle/ High (>MYR4850)	4.37 [42]	472	231 (48.9)	241 (51.1)	
Having chronic diseases	No	61.2 (%) [43]	628	343 (54.6)	285 (45.4)	χ^2^ = 0.24, *p* = 0.646
	Yes	38.8 (%) [43]	147	77 (52.3)	70 (47.6)	
Past COVID-19 infection	No		511	275 (53.8)	236 (46.2)	χ^2^ = 0.09, *p* = 0.769
	Yes		264	145 (54.9)	119 (45.1)	
Work from home (*n* = 755)	No		401	209 (52.1)	192 (47.9)	χ^2^ = 1.65, *p* = 0.200
	Yes		354	201 (56.8)	153 (43.2)	

^a^ Total population of Malaysia: 32.7 million. ^b^ Those who scored 22 or higher on the FAS. MYR 1 = USD 0.23 (19 February 2023). * Significant at *p* < 0.05.

**Table 2:** Association between mental health (DAS) score and fatigued and non-fatigued groups, *n* = 775.

Variable	B	Crude Odds Ratio	95% CI	*p*-Value
Depression score	0.25	1.29	1.24, 1.34	<0.001 *
Anxiety score	0.24	1.27	1.22, 1.32	<0.001 *
Stress score	0.21	1.23	1.12, 1.27	<0.001 *

* Significant at *p* < 0.05, using simple logistic regression.

**Table 3:** Association between various domains of perceived causes of pandemic fatigue and fatigued and non-fatigued groups, *n* = 775.

Variable	B	Crude Odds Ratio	95% CI	*p*-Value
Perception of fatigue to comply with the SOP of COVID-19	0.02	1.02	1.00, 1.04	0.018 *
Perception risk of infection with COVID-19	0.06	1.06	1.02, 1.11	0.008 *
Perception of distress due to the pandemic	0.16	1.17	1.12, 1.22	<0.001 *
Perception of negligence toward pandemic	0.10	1.10	1.05, 1.17	<0.001 *
Perception of change due to pandemic	0.11	1.12	1.06, 1.18	<0.001 *

* Significant at *p* < 0.05, using simple logistic regression.

## Data Availability

All relevant data are within the paper.

## References

[1] Wu Z., McGoogan J.M. (2020). Characteristics of and Important Lessons from the Coronavirus Disease 2019 (COVID-19) Outbreak in China: Summary of a Report of 72,314 Cases from the Chinese Center for Disease Control and Prevention. JAMA.

[2] Xu X.W., Wu X.X., Jiang X.G., Xu K.J., Ying L.J., Ma C.L., Li S.B., Wang H.Y., Zhang S., Gao H.N. (2020). Clinical findings in a group of patients infected with the 2019 novel coronavirus (SARS-Cov-2) outside of Wuhan, China: Retrospective case series. BMJ.

[3] Jee Y. (2020). WHO International Health Regulations Emergency Committee for the COVID-19 outbreak. Epidemiol. Health.

[4] Global Research Collaboration for Infectious Disease Preparedness (2020). COVID 2019 PHEIC Global Research and Innovation Forum: Towards a Research Roadmap.

[5] WHO WHO Director-General’s Opening Remarks at the Media Briefing on COVID-19 11 March 2020. https://www.who.int/director-general/speeches/detail/who-director-general-s-opening-remarks-at-the-media-briefing-on-covid-19---11-march-2020.

[6] WHO Coronavirus Disease (COVID-19) Pandemic. https://www.who.int/emergencies/diseases/novel-coronavirus-2019?adgroupsurvey={adgroupsurvey}&gclid=Cj0KCQiAg_KbBhDLARIsANx7wAz2vfleJym2JXoOXw2Wsxi-BByC053V_e-JV_Yi-jcb9ukdBKcdJQoaAq2kEALw_wcB.

[7] Aziz N.A., Othman J., Lugova H., Suleiman A. (2020). Malaysia’s approach in handling COVID-19 onslaught: Report on the Movement Control Order (MCO) and targeted screening to reduce community infection rate and impact on public health and economy. J. Infect. Public Health.

[8] Brauner J.M., Mindermann S., Sharma M., Johnston D., Salvatier J., Gavenčiak T., Stephenson A.B., Leech G., Altman G., Mikulik V. (2021). Inferring the effectiveness of government interventions against COVID-19. Science.

[9] Ministry of Health, Malaysia (2023). KKMNOW: COVID-19. In The Latest Data on the Pandemic in Malaysia. https://data.moh.gov.my/covid.

[10] Herng L.C., Singh S., Sundram B.M., Zamri A.S.S.M., Vei T.C., Aris T., Ibrahim H., Abdullah N.H., Dass S.C., Gill B.S. (2022). The effects of super spreading events and movement control measures on the COVID-19 pandemic in Malaysia. Sci. Rep..

[11] Kaos J. (2022). PM: Malaysia Will Transition into Endemic Phase from 1 April. The Star.

[12] Tullett W., McCann H. (2022). Sensing the pandemic: Revealing and re-ordering the senses. Senses Soc..

[13] Luo W., Liu Z., Zhou Y., Zhao Y., Li Y.E., Masrur A., Yu M. (2022). Investigating Linkages Between Spatiotemporal Patterns of the COVID-19 Delta Variant and Public Health Interventions in Southeast Asia: Prospective Space-Time Scan Statistical Analysis Method. JMIR Public Health Surveill.

[14] Xie Q., Sundararaj V., MR R. (2022). Analyzing the factors affecting the attitude of public toward lockdown, institutional trust, and civic engagement activities. J. Community Psychol..

[15] Abdul Aziz A.R., Ali Z., Mohd Noor N., Sulaiman S. (2021). Exploring the Compliance Behaviour During COVID-19 Pandemic from Social Psychology Perspectives. Int. J. Acad. Res. Bus. Soc. Sci..

[16] Maslach C., Leiter M.P. (2016). Understanding the burnout experience: Recent research and its implications for psychiatry. World Psychiatr..

[17] Bianchi R., Truchot D., Laurent E., Brisson R., Schonfeld I.S. (2014). Is burnout solely job-related? A critical comment. Scand. J. Psychol..

[18] American Psychiatric Association (2013). Diagnostic and Statistical Manual of Mental Disorders: DSM-5.

[19] Vindegaard N., Benros M.E. (2020). COVID-19 pandemic and mental health consequences: Systematic review of the current evidence. Brain Behav. Immun..

[20] Dai H., Zhang S.X., Looi K.H., Su R., Li J. (2020). Perception of Health Conditions and Test Availability as Predictors of Adults’ Mental Health during the COVID-19 Pandemic: A Survey Study of Adults in Malaysia. Int. J. Environ. Res. Public Health.

[21] Esterwood E., Saeed S.A. (2020). Past Epidemics, Natural Disasters, COVID19, and Mental Health: Learning from History as we Deal with the Present and Prepare for the Future. Psychiatr. Q..

[22] National Center for Immunization and Respiratory Diseases (NCIRD), Division of Viral Diseases Middle East Respiratory Syndrome (MERS). https://www.cdc.gov/coronavirus/mers/index.html.

[23] Jeong H., Yim H.W., Song Y.J., Ki M., Min J.A., Cho J., Chae J.H. (2016). Mental health status of people isolated due to Middle East Respiratory Syndrome. Epidemiol. Health.

[24] Morganstein J.C., Ursano R.J. (2020). Ecological Disasters and Mental Health: Causes, Consequences, and Interventions. Front. Psychiatr..

[25] Zeng N., Zhao Y.-M., Yan W., Li C., Lu Q.-D., Liu L., Ni S.-Y., Mei H., Yuan K., Shi L. (2023). A systematic review and meta-analysis of long term physical and mental sequelae of COVID-19 pandemic: Call for research priority and action. Mol. Psychiatr..

[26] Liu S., Haucke M.N., Heinzel S., Heinz A. (2021). Long-Term Impact of Economic Downturn and Loneliness on Psychological Distress: Triple Crises of COVID-19 Pandemic. J. Clin. Med..

[27] Dragioti E., Li H., Tsitsas G., Lee K.H., Choi J., Kim J., Choi Y.J., Tsamakis K., Estradé A., Agorastos A. (2022). A large-scale meta-analytic atlas of mental health problems prevalence during the COVID-19 early pandemic. J. Med. Virol..

[28] Robinson E., Sutin A.R., Daly M., Jones A. (2022). A systematic review and meta-analysis of longitudinal cohort studies comparing mental health before versus during the COVID-19 pandemic in 2020. J. Affect. Disord..

[29] Sampogna G., Pompili M., Fiorillo A. (2022). Mental Health in the Time of COVID-19 Pandemic: A Worldwide Perspective. Int. J. Environ. Res. Public Health.

[30] WHO Pandemic Fatigue: Reinvigorating the Public to Prevent COVID-19: Policy Framework for Supporting Pandemic Prevention and Management: Revised Version November 2020. https://apps.who.int/iris/bitstream/handle/10665/337574/WHO-EURO-2020-1573-41324-56242-eng.pdf?sequence=1&isAllowed=y.

[31] MacIntyre C.R., Nguyen P.-Y., Chughtai A.A., Trent M., Gerber B., Steinhofel K., Seale H. (2021). Mask use, risk-mitigation behaviours and pandemic fatigue during the COVID-19 pandemic in five cities in Australia, the UK and USA: A cross-sectional survey. Int. J. Infect. Dis..

[32] Taylor S., Rachor G.S., Asmundson G.J.G. (2022). Who develops pandemic fatigue? Insights from Latent Class Analysis. PLoS ONE.

[33] Yan E., Ng H.K.L., Lai D.W.L., Lee V.W.P. (2022). Physical, psychological and pandemic fatigue in the fourth wave of COVID-19 outbreak in Hong Kong: Population-based, cross-sectional study. BMJ Open.

[34] WHO WHO/Europe Discusses How to Deal with Pandemic Fatigue. https://www.who.int/news-room/feature-stories/detail/who-europe-discusses-how-to-deal-with-pandemic-fatigue.

[35] Rahimian Aghdam S., Alizadeh S.S., Rasoulzadeh Y., Safaiyan A. (2019). Fatigue Assessment Scales: A comprehensive literature review. Arch. Hyg. Sci..

[36] Michielsen H.J., De Vries J., Van Heck G.L. (2003). Psychometric qualities of a brief self-rated fatigue measure: The Fatigue Assessment Scale. J. Psychosom. Res..

[37] Lovibond P.F., Lovibond S.H. (1995). The structure of negative emotional states: Comparison of the Depression Anxiety Stress Scales (DASS) with the Beck Depression and Anxiety Inventories. Behav. Res. Ther..

[38] Krejcie R.V., Morgan D.W. (1970). Determining sample size for research activities. Educ. Psychol. Meas..

[39] Drent M., Lower E.E., De Vries J. (2012). Sarcoidosis-associated fatigue. Eur. Respir. J..

[40] Musa R., Fadzil M.A., Zain Z. (2007). Translation, validation and psychometric properties of Bahasa Malaysia version of the Depression Anxiety and Stress Scales (DASS). ASEAN J. Psychiatr..

[41] Prime Minister’S Department, Department Of Statistics Malaysia (2022). Press Release Current Population Estimates, Malaysia. https://www.dosm.gov.my/v1/index.php?r=column/pdfPrev&id=dTZXanV6UUdyUEQ0SHNWOVhpSXNMUT09.

[42] Department Of Statistics Malaysia Press Release. Household Income & Basic Amenities Survey Report 2019. https://www.dosm.gov.my/v1/index.php?r=column/cthemeByCat&cat=120&bul_id=TU00TmRhQ1N5TUxHVWN0T2VjbXJYZz09&menu_id=amVoWU54UTl0a21NWmdhMjFMMWcyZz09#.

[43] National Health and Morbidity Survey (NHMS) (2019). Vol. I: NCDs—Non-Communicable Diseases: Risk Factors and Other Health Problems. https://iku.gov.my/images/IKU/Document/REPORT/NHMS2019/Report_NHMS2019-NCD_v2.pdf.

[44] Tabari P., Amini M., Moghadami M., Moosavi M. (2020). International Public Health Responses to COVID-19 Outbreak: A Rapid Review. Iran. J. Med. Sci..

[45] Morgul E., Bener A., Atak M., Akyel S., Aktaş S., Bhugra D., Ventriglio A., Jordan T.R. (2020). COVID-19 pandemic and psychological fatigue in Turkey. Int. J. Soc. Psychiatr..

[46] Uygur O.F., Uygur H. (2021). Association of post-COVID-19 fatigue with mental health problems and sociodemographic risk factors. Fatigue Biomed. Health Behav..

[47] Leung H.T., Gong W.-J., Sit S.M.M., Lai A.Y.K., Ho S.Y., Wang M.P., Lam T.H. (2022). COVID-19 pandemic fatigue and its sociodemographic and psycho-behavioral correlates: A population-based cross-sectional study in Hong Kong. Sci. Rep..

[48] Xin L., Wang L., Cao X., Tian Y., Yang Y., Wang K., Kang Z., Zhao M., Feng C., Wang X. (2022). Prevalence and influencing factors of pandemic fatigue among Chinese public in Xi’an city during COVID-19 new normal: A cross-sectional study. Front. Public Health.

[49] Santomauro D.F., Mantilla Herrera A.M., Shadid J., Zheng P., Ashbaugh C., Pigott D.M., Abbafati C., Adolph C., Amlag J.O., Aravkin A.Y. (2021). Global prevalence and burden of depressive and anxiety disorders in 204 countries and territories in 2020 due to the COVID-19 pandemic. Lancet.

[50] Knolle F., Ronan L., Murray G.K. (2021). The impact of the COVID-19 pandemic on mental health in the general population: A comparison between Germany and the UK. BMC Psychol..

[51] Shi L., Lu Z.A., Que J.Y., Huang X.L., Liu L., Ran M.S., Gong Y.M., Yuan K., Yan W., Sun Y.K. (2020). Prevalence of and Risk Factors Associated with Mental Health Symptoms Among the General Population in China During the Coronavirus Disease 2019 Pandemic. JAMA Netw. Open.

[52] Department of Statistics Malaysia (2020). Household Income Estimates and Incidence of Poverty Report, Malaysia. https://www.dosm.gov.my/v1/index.php?r=column/cthemeByCat&cat=493&bul_id=VTNHRkdiZkFzenBNd1Y1dmg2UUrZz09&menu_id=amVoWU54UTl0a21NWmdhMjFMMWcyZz09#:~:text=In%202020%2C%20there%20was%20an,moved%20to%20the%20B40%20group.

[53] Wong L.P., Alias H., Md Fuzi A.A., Omar I.S., Mohamad Nor A., Tan M.P., Baranovich D.L., Saari C.Z., Hamzah S.H., Cheong K.W. (2021). Escalating progression of mental health disorders during the COVID-19 pandemic: Evidence from a nationwide survey. PLoS ONE.

[54] Tang K.H.D. (2022). Movement control as an effective measure against COVID-19 spread in Malaysia: An overview. J. Public Health.

[55] Ismail M.K., Sarifuddin S., Muhamad M.Z., Siwar C. (2022). Levels of Stress, Anxiety, and Depression in the Initial Stage of Movement Control Order in Malaysia: A Sociodemographic Analysis. Proceedings.

[56] Haktanir A., Can N., Seki T., Kurnaz M.F., Dilmaç B. (2021). Do we experience pandemic fatigue? current state, predictors, and prevention. Curr. Psychol..

[57] Kesebir P. (2014). A quiet ego quiets death anxiety: Humility as an existential anxiety buffer. J. Personal. Soc. Psychol..

[58] Zhang J., Peng J., Gao P., Huang H., Cao Y., Zheng L., Miao D. (2019). Relationship between meaning in life and death anxiety in the elderly: Self-esteem as a mediator. BMC Geriatr..

[59] Menzies R.E., Menzies R.G. (2020). Death anxiety in the time of COVID-19: Theoretical explanations and clinical implications. Cogn. Behav. Ther..

[60] Bulut M.B. (2022). Relationship between COVID-19 anxiety and fear of death: The mediating role of intolerance of uncertainty among a Turkish sample. Curr. Psychol..

[61] Chalhoub Z., Koubeissy H., Fares Y., Abou-Abbas L. (2022). Fear and death anxiety in the shadow of COVID-19 among the Lebanese population: A cross-sectional study. PLoS ONE.

[62] Bahar Moni A.S., Abdullah S., Abdullah M.F.I.L., Kabir M.S., Alif S.M., Sultana F., Salehin M., Islam S.M.S., Cross W., Rahman M.A. (2021). Psychological distress, fear and coping among Malaysians during the COVID-19 pandemic. PLoS ONE.

[63] Taylor S. (2022). The Psychology of Pandemics. Annu. Rev. Clin. Psychol..

[64] Leavy R.L. (1983). Social support and psychological disorder: A review. J. Community Psychol..

[65] Kessler R.C., Price R.H., Wortman C.B. (1985). Social factors in psychopathology: Stress, social support, and coping processes. Annu. Rev. Psychol..

[66] McGinty E.E., Presskreischer R., Han H., Barry C.L. (2020). Psychological Distress and Loneliness Reported by US Adults in 2018 and April 2020. JAMA.

[67] Saltzman L.Y., Hansel T.C., Bordnick P.S. (2020). Loneliness, isolation, and social support factors in post-COVID-19 mental health. Psychol. Trauma Theory Res. Pract. Policy.

[68] Alnazly E., Khraisat O.M., Al-Bashaireh A.M., Bryant C.L. (2021). Anxiety, depression, stress, fear and social support during COVID-19 pandemic among Jordanian healthcare workers. PLoS ONE.

[69] Ministry of Health, Malaysia National COVID-19 Testing Strategy [Press Release]. https://covid-19.moh.gov.my/reopeningsafely/nts.

[70] Esa M., Ibrahim F.S., Mustafa Kamal E. (2020). COVID-19 pandemic lockdown: The consequences towards project success in Malaysian construction industry. Adv. Sci. Technol. Eng. Syst. J..

[71] Piraveenan M., Sawleshwarkar S., Walsh M., Zablotska I., Bhattacharyya S., Farooqui H.H., Bhatnagar T., Karan A., Murhekar M., Zodpey S. (2021). Optimal governance and implementation of vaccination programmes to contain the COVID-19 pandemic. R. Soc. Open Sci..

[72] Md Iderus N.H., Lakha Singh S.S., Mohd Ghazali S., Yoon Ling C., Cia Vei T., Md Zamri A.S.S., Ahmad Jaafar N., Ruslan Q., Ahmad Jaghfar N.H., Gill B.S. (2022). Correlation between Population Density and COVID-19 Cases during the Third Wave in Malaysia: Effect of the Delta Variant. Int. J. Environ. Res. Public Health.

[73] Law L.N.-S., Loo K.-Y., Goh J.X.H., Pusparajah P. (2021). Omicron: The rising fear for another wave in Malaysia. Prog. Microbes Mol. Biol..

[74] Petherick A., Goldszmidt R., Andrade E.B., Furst R., Hale T., Pott A., Wood A. (2021). A worldwide assessment of changes in adherence to COVID-19 protective behaviours and hypothesized pandemic fatigue. Nat. Hum. Behav..

[75] Rashid A.A., Rashid M.R.A., Yaman M.N., Mohamad I. (2020). Teaching Medicine Online During the COVID-19 Pandemic: A Malaysian Perspective. Bangladesh J. Med. Sci..

[76] Abiddin N.Z., Ibrahim I., Abdul Aziz S.A. (2022). A Literature Review of Work From Home Phenomenon During COVID-19 Toward Employees’ Performance and Quality of Life in Malaysia and Indonesia. Front. Psychol..

[77] Van Loenhout J.A.F., Vanderplanken K., Scheen B., Van den Broucke S., Aujoulat I. (2021). Determinants of adherence to COVID-19 measures among the Belgian population: An application of the protection motivation theory. Arch. Public Health.

[78] Dekeyser S., Schmits E., Glowacz F., Klein O., Schmitz M., Wollast R., Yzerbyt V., Luminet O. (2023). Predicting Compliance with Sanitary Behaviors among Students in Higher Education During the Second COVID-19 Wave: The Role of Health Anxiety and Risk Perception. Psychol. Belg..

[79] Supriyati Supriyati D., Fahmi Baiquni M.P.H., Tri Siswati D., Herni Endah Widyawati S.T., Gz R., Rahmawati S.T.P., Wardani R.K., Gz S. (2022). Social determinants of health protocol adherence among adults during COVID-19 pandemic in Yogyakarta, Indonesia. Med. J. Malays..

[80] Ojo A.O., Arasanmi C.N., Raman M., Tan C.N.-L. (2019). Ability, motivation, opportunity and sociodemographic determinants of Internet usage in Malaysia. Inf. Dev..

[81] Kyaw T.M., Deng A.G., Mano Mohen S., Uvaraja V.D., Mustafa S.M. (2022). Assessment of Digital Health Literacy and Its Associated Factors Among University Students During COVID-19 Pandemic in Malaysia. J. Health Lit..

[82] Selvaraj A., Radhin V., Ka N., Benson N., Mathew A.J. (2021). Effect of pandemic based online education on teaching and learning system. Int. J. Educ. Dev..

[83] Harun Z., Hamzah F.M., Mansor S., Mahmud A.S., Hashim H., Sultan M.T.H., Mohamed N.M.Z.N., Ibrahim M.D., Hasin H., Saad M.R. (2021). COVID-19 Effects on Students’ Teaching and Learning Perspectives in Malaysian Varsities. Pertanika J. Soc. Sci. Humanit..

[84] Bozkurt V. (2020). Working during a pandemic: Economic concerns, digitalization, and productivity. COVID-19 Pandemic Its Econ. Soc. Political Impacts.

[85] Banerjee D., Vaishnav M., Rao T.S., Raju M., Dalal P., Javed A., Saha G., Mishra K.K., Kumar V., Jagiwala M.P. (2020). Impact of the COVID-19 pandemic on psychosocial health and well-being in South-Asian (World Psychiatric Association zone 16) countries: A systematic and advocacy review from the Indian Psychiatric Society. Indian J. Psychiatry.

[86] Ministry of Finance, Malaysia Budget Speech 2023. https://budget.mof.gov.my/pdf/2023/ucapan/buku-budget-speech-2023.pdf.

